# Supplementary material for: Evidence of exciton-libron coupling in chirally adsorbed single molecules
Source: Nat Commun. 2022 Oct 12;13:6008. doi: 10.1038/s41467-022-33653-7 (PMC9556530; doi:10.1038/s41467-022-33653-7)
Supplement: Supplementary file 1 — Supplementary Information [file 41467_2022_33653_MOESM1_ESM.pdf]

Supplementary Information

Evidence of exciton-libron coupling in chirally adsorbed single molecules

Jiří Doležal[1,2]*, Sofia Canola[1], Prokop Hapala[1], Rodrigo Cezar de Campos Ferreira[1], Pablo Merino[3,4], Martin Švec[1,5]*

[1] Institute of Physics, Czech Academy of Sciences; Cukrovarnická 10/112, CZ16200 Praha 6, Czech Republic

[2] Faculty of Mathematics and Physics, Charles University; Ke Karlovu 3, CZ12116 Praha 2, Czech Republic

[3] Catalan Institute of Nanoscience and Nanotechnology (ICN2), CSIC and BIST, Campus UAB, Bellaterra, E08193 Barcelona, Spain

[4] Instituto de Ciencia de Materiales de Madrid; CSIC, Sor Juana Inés de la Cruz 3, E28049 Madrid, Spain

[5] Institute of Organic Chemistry and Biochemistry, Czech Academy of Sciences; Flemingovo náměstí 542/2. CZ16000 Praha 6, Czech Republic

corresponding authors: dolezalj@fzu.cz, svec@fzu.cz

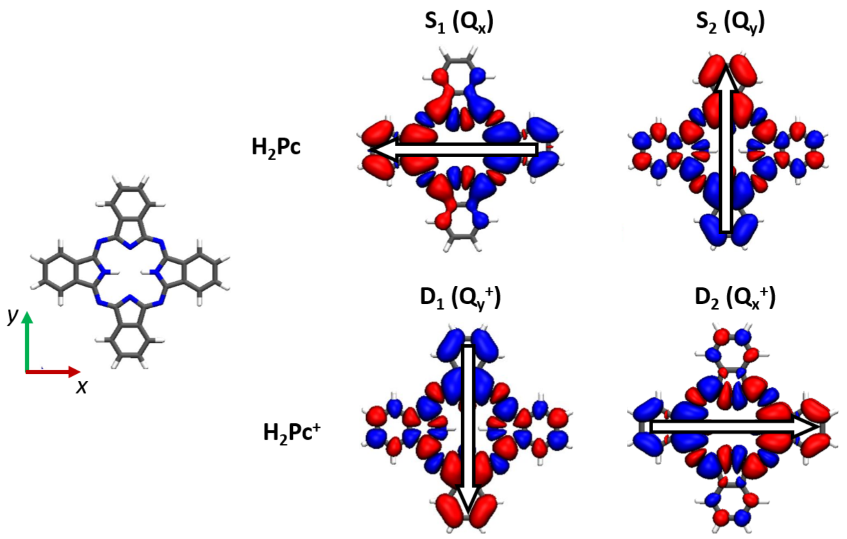

Supplementary Figure. 1: *Transition densities and transition dipole moments (white arrow) of the first two excited states of H_2Pc neutral (S_1 and S_2 states) and cation (D_1 and D_2 states), see Supplementary Table 1. Calculations TD(TDA)-wB97XD/6-31G*. Isosurface 0.002 au.*

	**n.state(sym)**	**Exc/eV (osc.)**	**band** [a]	**exp.**
**H_2Pc neutral**	S_1 (B1u)	1.89 (0.472)	Q_x	1.80
	S_2 (B2u)	2.04 (0.542)	Q_y	1.93
**H_2Pc+ cation**	D_1 (B2u)	1.50 (0.241)	Q_y^+	1.39
	D_2 (B1u)	1.71 (0.172)	Q_x^+	-
**ZnPc neutral**	S_1	1.92 (0.510)	Q	1.89
**ZnPc+ cation**	D_1	1.63 (0.205)	Q^+	1.52
**MgPc neutral**	S_1	1.88 (0.502)	Q	1.89
**MgPc+ cation**	D_1	1.59 (0.205)	Q^+	1.51

[a] For H_2Pc, *x* axis oriented along N-H …. H-N direction, see Supplementary Figure 1.

Supplementary Table 1: *Calculations TD- (TDA-)wB97XD/6-31G* of H_2Pc, ZnPc, MgPc neutral (cation): state number (with symmetry label), emission energy, oscillator strengths and band assignment. Comparison with experimental excitation energies taken from the spectra of Fig. 1.*

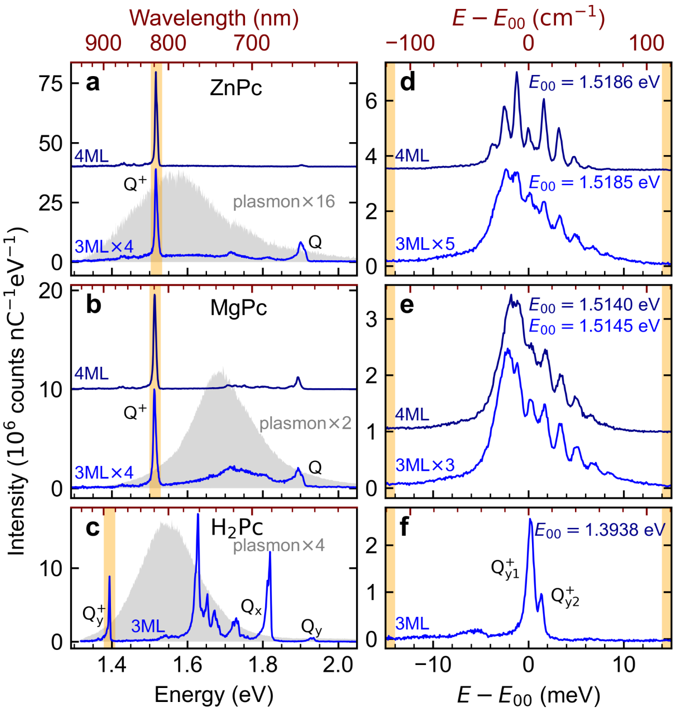

Supplementary Figure 2: *a-c) Raw overview STM-EL spectra from Figure 1 of the ZnPc, MgPc and H_2Pc at -2.8 V, 100 pA, showing the neutral (Q, Q_x, Q_y) and cation (Q^+, Q_y^+) emission fingerprints. Grey-filled spectra on the background of each panel are the responses of the nanocavities measured on a clean Ag(111) surface at 2.5 V, 1 nA. d-f) Raw spectra measured at the same bias and tunnelling current with 400 µeV resolution on the ZnPc, MgPc and H_2Pc cations, respectively, evidencing the fine structure present in the first two cases. The scale is given relative to the central peaks in the spectral manifold, which manifest lower intensity with respect to their neighbours. The reference energies E_{00} are set to the assumed zero phonon lines in each spectrum. Source data are provided as a Source Data file.*

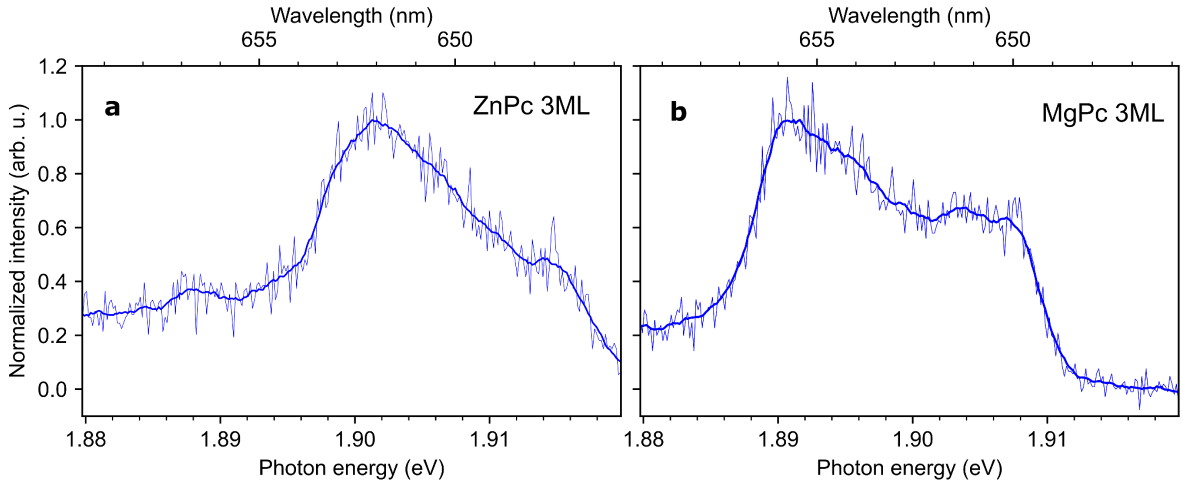

Supplementary Figure 3: *High-resolution STM-EL spectra measured at the lobe position corresponding to the neutral Q peak of ZnPc in a) and MgPc molecule in b) on 3 ML NaCl.*

Acquisition parameters: energy resolution 600 μeV, a) U_S = -2.8 V , t = 60 s , I = 133 pA, b) U_S= -2.8 V, t = 60 s, I = 100 pA. The estimated FWHM is 13 meV in a) and 20 meV in b).

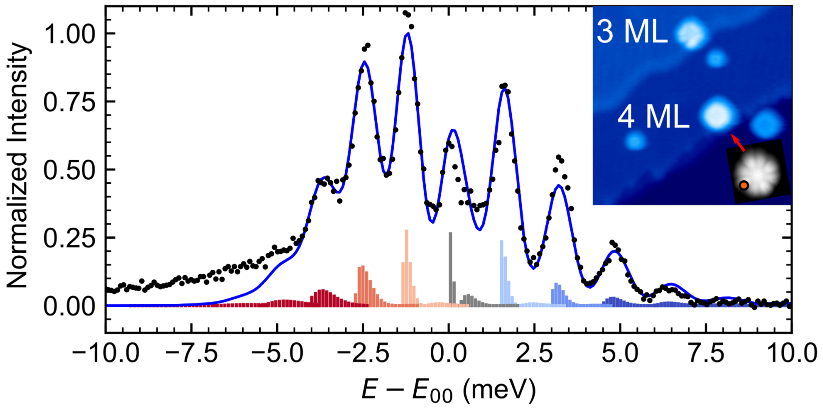

Supplementary Figure 4: *Experimental (black dotted, U_S = -2.9 V , t = 180 s , I = 20 pA) and simulated (solid blue line) STM-EL fine spectrum of Q^+ peak of chirally adsorbed step-edge stabilized $MgPc^+$ on 4ML NaCl/Au(111). The Franck-Condon factors are calculated including the modified exponential distribution and are colour-coded according to the vibration quantum number difference between the initial and final state, i.e. m - n (red - negative, blue - positive, grey - null). The energies E_{00} obtained through the fitting are set as the reference in each spectrum. Parameters of the simulated spectrum: k_0 = 1.60 meV/(°)^2, k_1 = 1.86 meV/(°)^2, $\Delta\phi_0$= 0.685°, T_{eff} = 63 K, A = 0.68, γ = 0.65 meV, E_{00} = 1.5144 eV.*

	**cation**			**neutral**		
	k_0 **(meV/(°)^2)**	***k_1*** **(meV/(°)^2)**	**$\Delta\phi_0$(°)**	***k_0*** **(meV/(°)^2)**	***k_1*** **(meV/(°)^2)**	**$\Delta\phi_0$(°)**
ZnPc	1.769	1.904	0.31	1.459	1.527	0.42
MgPc	1.747	1.880	0.28	1.461	1.535	0.41
H_2Pc	1.569	1.634	0.00	1.633	1.675	0.00

Supplementary Table 2**:** Parameters of parabolic fitting of total energies computed by TDDFT (see also Fig. 2 and Supplementary Figure 6).

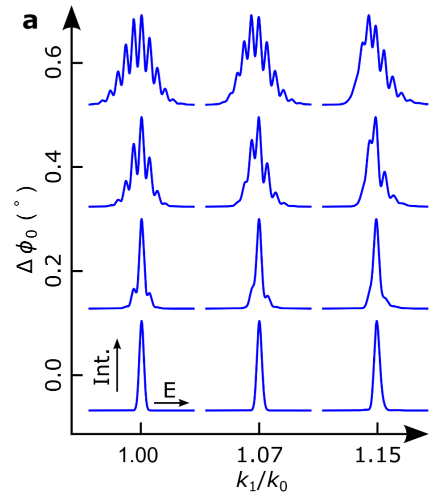

Supplementary Figure 5: *Typology of simulated STM-EL spectra for varying values of excited/ground state equilibrium angle* $\Delta\boldsymbol{\phi}_0$*, potential stiffness ratio* k_1/k_0 *and reduction factor A=1 in a) and A=0.5 in b) (using T = 70 K,* γ *= 0.83 meV ,* k_1 *= 1.83 meV/(°)²* *and J = 113* m_p *nm²). In the limiting case of* $\Delta\boldsymbol{\phi}_0$ *= 0 and* k_1/k_0 *= 1, the spectrum consists of a single peak resulting from the sum of all m = n transitions. Increasing* $\Delta\boldsymbol{\phi}_0$ *> 0 leads to significant overlap among different initial and final librational states (m - n ≠ 0) and consequent appearance of the red- and blue-shifted peak progressions. The ratio* $k_1/k_0 \neq 1$ *affects the asymmetry of the spectral envelope around* E_{00} *and the energy spacings among the peaks. For* k_1/k_0 *> 1, the interpeak energy difference in the red-shifted branch becomes smaller compared to the blue-shifted branch of the spectrum.*

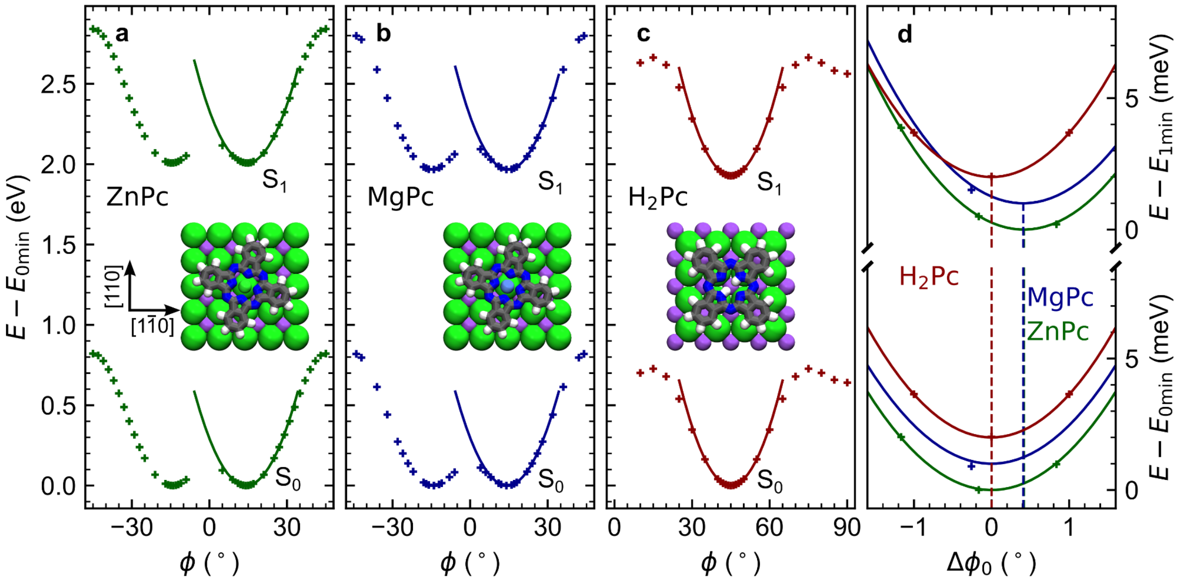

Supplementary Figure 6: *Total energy as a function of rotation by angle* $\boldsymbol{\phi}$ *for the ground and excited states of a) ZnPc, b) MgPc and c)* H_2Pc*: computed energy is plotted with points and the corresponding parabolic fits around the local minima with solid lines. The insets show the schematic models of the respective ground state neutral molecules in their*

equilibrium positions, i.e. rotated ~15° centred above Cl- in the case of ZnPc and MgPc, and rotated 45° above Na^+ in the case of H_2Pc. The angle $\boldsymbol{\phi}$ is defined as between the molecule x-axes (crossing two opposing isoindole groups along N - N atom direction) and the [110] NaCl direction. d) The detailed comparison of the potential well minima of the three neutral chromophores as a function of the shift in the equilibrium angle positions $\Delta\boldsymbol{\phi}_0$ between the ground and excited states. MgPc and H_2Pc ground and excited state are vertically offset by 1 and 2 meV for clarity. Note the zero shift for H_2Pc, dictated by the symmetry of the system. Fitting parameters are summarized in Supplementary Table 2.
